# Two new glassfrogs (Centrolenidae: *Hyalinobatrachium*) from Ecuador, with comments on the endangered biodiversity of the Andes

**DOI:** 10.7717/peerj.13109

**Published:** 2022-03-18

**Authors:** Juan M. Guayasamin, Rebecca M. Brunner, Anyelet Valencia-Aguilar, Daniela Franco-Mena, Eva Ringler, Anderson Medina Armijos, Carlos Morochz, Lucas Bustamante, Ross J. Maynard, Jaime Culebras

**Affiliations:** 1 Laboratorio de Biología Evolutiva, Instituto Biósfera, Colegio de Ciencias Biológicas y Ambientales COCIBA, Universidad San Francisco de Quito USFQ, Quito, Ecuador; 2 Department of Biology, University of North Carolina at Chapel Hill, Chapel Hill, North Carolina, United States; 3 Third Millennium Alliance, Quito, Ecuador; 4 Department of Environmental Science, Policy, and Management, University of California, Berkeley, Berkeley, California, United States; 5 Division of Behavioral Ecology, Institute of Ecology and Evolution, University of Bern, Bern, Switzerland; 6 Laboratorio de Biología Evolutiva, Instituto Biósfera, Colegio de Ciencias Biológicas y Ambientales, Universidad San Francisco de Quito USFQ, Quito, Ecuador; 7 Biology & Research Department, Mashpi Lodge, Mashpi, Ecuador; 8 Tropical Herping, Quito, Ecuador; 9 The Biodiversity Group, Tucson, Arizona, United States; 10 Photo Wildlife Tours, Quito, Ecuador; 11 Fundación Cóndor Andino, Quito, Ecuador

**Keywords:** Andes, Amphibia, Conservation, Cryptic diversity, Mining, Ecuador

## Abstract

**Background:**

The Tropical Andes is the world’s most biodiverse hotspot. This region contains >1,000 amphibian species, more than half of which are endemic. Herein we describe two new glassfrog species (Centrolenidae: *Hyalinobatrachium*) that we discovered within relatively unexplored and isolated localities of the Ecuadorian Andes.

**Methods:**

We employed morphological, acoustic, and molecular methods to test the hypothesis that *Hyalinobatrachium mashpi* sp. nov and *H. nouns* sp. nov. are species new to science. Following standard methods, we generated mitochondrial sequences (16S) of 37 individuals in the genus *Hyalinobatrachium*. We inferred the phylogenetic relationships of the two new species in comparison to all other glassfrogs using Maximum Likelihood. In addition to describing the call of *H. mashpi* sp. nov., we performed a discriminant analysis of principal components (DAPC) with the advertisement call characteristics of several congeners.

**Results:**

Based on an integrative taxonomy approach, we describe two new species. Morphological traits and the inferred phylogeny unambiguously place the new taxa in the genus *Hyalinobatrachium*. Both species are distinguished from other glassfrogs mainly by their dorsal coloration (*i.e*., dorsum lime green with small light yellow spots, head usually with interorbital bar) and transparent pericardium (*i.e*., the heart is visible through the ventral skin). The new species exhibit a high morphological similarity (*i.e*., cryptic) and occur within relatively close geographical proximity (closest aerial distance = 18.9 km); however, their uncorrected *p* distance for the mitochondrial gene 16S is 4.6–4.7%, a value that greatly exceeds the genetic distance between closely related species of centrolenid frogs. The DAPC revealed that the advertisement call of *H. mashpi* sp. nov. is acoustically distinct.

**Discussion:**

Our findings are congruent with several previous studies that report a high degree of endemism in the Toisán mountain range, which appears to be isolated from the main Andean cordillera for some amphibian groups. We recommend that both *H. mashpi* sp. nov. and *H. nouns* sp. nov. be listed as Endangered, following IUCN criteria. These new species provide another example of cryptic diversity in the Andes—further evidence that the region fosters much more biodiversity than we have the resources to catalog. Threatened by mining and other exploitative industries, these glassfrogs and many other yet-to-be-discovered Andean species highlight the dire need for effective conservation measures—especially in northwestern Ecuador.

## Introduction

The diversity of glassfrogs (Family Centrolenidae) is concentrated in the northern Andes, which hosts more than half (83 taxa) of the species in the family (Guayasamin et al., 2020). The linearity of the Andes, combined with its topographical and climatic complexity, has facilitated numerous diversification events—dominated by allopatric speciation, niche conservatism, and few ecological shifts (Hutter, Guayasamin & Wiens, 2013; Castroviejo-Fisher et al., 2014; Guayasamin et al., 2020). As a consequence, glassfrogs tend to occupy narrow distribution ranges in this biogeographic region, often restricted by elevation and river valleys (Guayasamin et al., 2020).

Within Centrolenidae, *Hyalinobatrachium* is particularly charismatic due to its peculiar morphological and behavioral traits. All species in the genus have ventral transparency (Ruiz-Carranza & Lynch, 1991; Cisneros-Heredia & Mcdiarmid, 2007; Guayasamin et al., 2009) and extended paternal care⁠—a derived trait that has evolved at least twice in the family (Delia, Bravo-Valencia & Warkentin, 2017). Although *Hyalinobatrachium* species have been the focus of numerous behavioral and ecological studies (Vockenhuber, Hödl & Karpfen, 2008; Delia et al., 2010; Mangold et al., 2015; Delia, Bravo-Valencia & Warkentin, 2017; Valencia-Aguilar, Guayasamin & Prado, 2021), their taxonomy is complex because they exhibit remarkable morphological conservatism (Castroviejo-Fisher et al., 2009, 2011; Guayasamin et al., 2009). Additionally, locating *Hyalinobatrachium* spp. in the Andean cloud forests is challenging, as they typically occupy high vegetation along steep streams and rivers. Our recent work in Andean localities of northwestern Ecuador has provided enough data to describe two new (and beautiful) glassfrog species. Because the habitat is severely fragmented and experiences constant deforestation and mining pressures, both species are of conservation concern.

## Materials and Methods

### Ethical statement

Research was conducted under permits MAE-DNB-CM-2015-0017, 019-2018-IC-FAU-DNB/MAE, and MAE-DNB-CM-2018-0105, issued by the Ministerio del Ambiente del Ecuador. The study was carried out in accordance with the guidelines for use of live amphibians and reptiles in field research (Beaupre et al., 2004), compiled by the American Society of Ichthyologists and Herpetologists (ASIH), the Herpetologists’ League (HL) and the Society for the Study of Amphibians and Reptiles (SSAR). We confirm that out study is reported in accordance with ARRIVE guidelines (https://arriveguidelines.org). Access to field sites was granted by Mashpi Reserve and Fundación Ecominga.

**Taxonomy and species concept.** Glassfrog taxonomy follows the proposal by Guayasamin et al. (2009). Species are considered separately evolving lineages, following the conceptual framework developed by Simpson (1951, 1961), Wiley (1978), and De Queiroz (2007). Determining if a given population is an independent lineage is a non-trivial task, and requires an integrative approach to assess species hypotheses (Dayrat, 2005; Padial et al., 2010).

**Figure 1:**
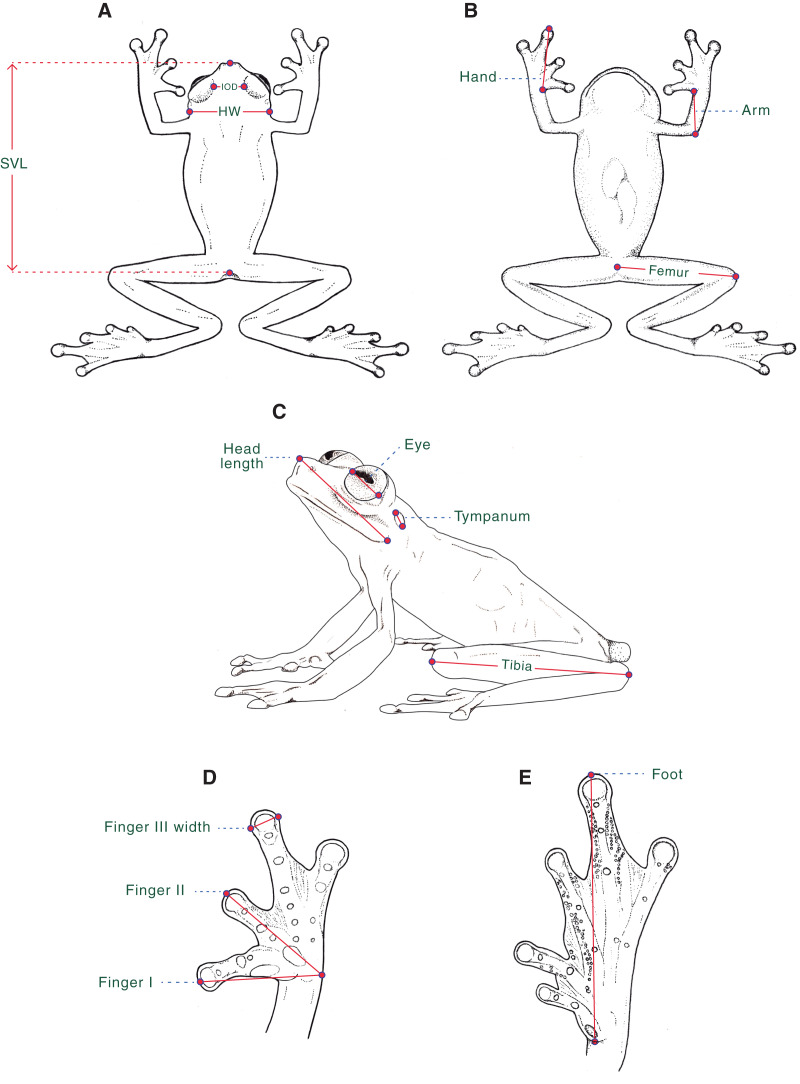
Morphological measurements as obtained in this study. Measurements are described in the text. SVL, Snout–vent length; HW, Head width; IOD, Interorbital distance. Ilustrations by Valentina Nieto Fernández.

**Morphological data.** For the diagnosis and description of the new species, we follow Lynch & Duellman (1973), Cisneros-Heredia & Mcdiarmid (2007), and Guayasamin et al. (2009). Webbing formula follows Savage & Heyer (1967), as modified by Guayasamin et al. (2006). We compared *Hyalinobatrachium* specimens housed at the following collections (Material S1): Centro Jambatu de Investigación y Conservación de Anfibios, Quito, Valle de San Rafael, Ecuador (CJ); Instituto de Ciencias Naturales, Universidad Nacional de Colombia, Bogotá, Colombia (ICN); University of Kansas, Museum of Natural History, Division of Herpetology, Lawrence, Kansas, USA (KU); Museo de Zoología, Universidad Tecnológica Indoamérica, Quito, Ecuador (MZUTI); National Museum of Natural History, Smithsonian Institution, Washington, D.C., USA (USNM); and Museo de Zoología, Universidad San Francisco de Quito, Quito, Ecuador (ZSFQ). We obtained morphological data with a Mitutoyo® digital caliper to the nearest 0.1 mm, as described below (Fig. 1): (1) snout–vent length (SVL) = distance from tip of snout to posterior margin of vent; (2) femur = distance from cloaca to knee; (3) tibia = length of flexed leg from knee to heel; (4) foot = distance from proximal edge of Toe I to tip of Toe IV ; (5) head length = distance from tip of snout to posterior angle of jaw articulation; (6) head width (HW) = width of head measured at level of jaw articulation; (7) interorbital distance (IOD) = shortest distance between upper eyelids, a measurement that equals to the subjacent frontoparietal bones; (8) eye = distance between anterior and posterior borders of the eye; (9) tympanum = distance between anterior and posterior borders of tympanic annulus; (10) arm = length of flexed forearm from elbow to proximal edge of Finger I at the level of articulation with arm; (11) hand = distance from proximal edge of Finger I to tip of Finger III; (12) Finger I = distance from outer margin of hand to tip of Finger I; (13) Finger II = distance from outer margin of hand to tip of Finger II; and (14) width of Finger III = maximum width of Finger III measured at distal end. We determined sexual maturity of examined frogs by the presence of vocal slits in museum specimens and calling activity in males during fieldwork.

**Evolutionary relationships.** We sequenced mitochondrial 16S in 37 individuals, including the two new taxa described below, as well as the morphologically similar *H. valerioi* (Dunn, 1931) and *H. aureoguttatum* (Barrera-Rodriguez & Ruiz-Carranza, 1989) (Material S2). Extraction, amplification, and sequencing protocols are described in Guayasamin et al. (2008) and Peñafiel et al. (2019). The obtained data were compared with homologous sequences from all available species in the genus *Hyalinobatrachium* and its sister taxon *Celsiella* (Guayasamin et al., 2008), downloaded from GenBank (https://www.ncbi.nlm.nih.gov/genbank/) and generated mostly by Guayasamin et al. (2008), Castroviejo-Fisher et al. (2014), and Twomey, Delia & Castroviejo-Fisher (2014). We also included data from the following newly described species: *H. yaku* Guayasamin et al., 2017a, *H. muiraquitan* De Oliveira & Hernández-Ruz, 2017, and *H. adespinosai* Guayasamin et al., 2019a. Sequences were aligned using MAFFT v.7 (Multiple Alignment Program for Amino Acid or Nucleotide Sequences: http://mafft.cbrc.jp/alignment/software/), with the Q-INS-i strategy. The software Mesquite (Maddison & Maddison, 2019) was used to visualize the alignment (no modifications were necessary). Maximum likelihood trees were estimated using GARLI 0.951 (Genetic Algorithm for Rapid Likelihood Inference; Zwickl, 2006). GARLI uses a genetic algorithm that finds the tree topology, branch lengths, and model parameters that maximize lnL simultaneously (Zwickl, 2006). Default values were used for other GARLI settings, as per recommendations of the developer (Zwickl, 2006). Bootstrap support was assessed *via* 1,000 pseudoreplicates under the same settings used in tree search. Genetic distances were calculated using PAUP* (Swofford, 2002).

**Bioacoustics.** We describe the call of the new *Hyalinobatrachium* species found in Mashpi and Tayra Reserves, as well as the vocalizations from morphologically and/or phylogenetically similar species: *Hyalinobatrachium adespinosai, H. aureoguttatum*, *H. chirripoi*, *H. pellucidum*, *H. tatayoi*, and *H. valerioi*. Calls of the new species were recorded with a Tascam DR-05; calls of *H. adespinosai*, *H. aureoguttatum*, *H. chirripoi*, *H. pellucidum*, and *H. tatayoi* were obtained with an Olympus LS-10 Linear PCM Field Recorder and/or a Roland R-26 digital recorder with a Sennheiser ME 67 directional microphone. All vocalizations were recorded in WAV format with a sampling rate of 44.1 kHz/s with 16 bits/sample. Recordings of *Hyalinobatrachium valerioi* by Roy McDiarmid in Costa Rica were obtained from the Macaulay Library (ML) of the Cornell Lab of Ornithology. We unfortunately were unable to record the new *Hyalinobatrachium* species from the Toisán Mountain Range, despite several attempts (*i.e*., males were not calling when located in the field). Measurements and definition of acoustic variables follow Köhler et al. (2017). Notes were divided into two classes—pulsed or tonal—based upon distinct waveforms in the oscillogram. Pulsed (or peaked) notes are defined as having one or more clear amplitude peak(s) and amplitude modulation (*i.e*., visible increases and decreases in amplitude on the oscillogram throughout the call); tonal notes are defined as having no clear amplitude peak (Dautel et al., 2011). To determine if major call characteristics (peak frequency, maximum frequency, minimum frequency, call duration, and inter-call duration) cluster by species, we performed a discriminant analysis of principal components (DAPC; Jombart, Devillard & Balloux, 2010), using the R package adegenet. DAPC maximizes differentiation between pre-defined groups (in this case, the new and related *Hyalinobatrachium* species listed above, except for *H. chirripoi*, due to lack of sufficient data), by transforming data *via* principal components analysis (PCA) and subsequently identifying clusters *via* discriminant analysis (DA).

New Zoological Taxonomic Names: The electronic version of this article in Portable Document Format (PDF) will represent a published work according to the International Commission on Zoological Nomenclature (ICZN), and hence the new names contained in the electronic version are effectively published under that Code from the electronic edition alone. This published work and the nomenclatural acts it contains have been registered in ZooBank, the online registration system for the ICZN. The ZooBank LSIDs (Life Science Identifiers) can be resolved and the associated information viewed through any standard web browser by appending the LSID to the prefix http://zoobank.org/. The LSID for this publication is:

urn:lsid:zoobank.org:pub:0C4888D5-2DB9-4421-A96E-7E41C17EC82F. The online version of this work is archived and available from the following digital repositories: PeerJ, PubMed Central SCIE and CLOCKSS.

## Results

**Evolutionary relationships**: The phylogeny (Fig. 1) confirms the placement of the two new species within the genus *Hyalinobatrachium* with significant support (bootstrap support = 96). The two new species show considerable genetic divergence (uncorrected *p* distance = 4.6–4.7% for the mitochondrial gene 16S), especially considering that the are found only 18.9 km apart (but with the Intag-Guayllabamba river valley between them). *Hyalinobatrachium mashpi* sp. nov. is sister to unidentified populations from Colombia (MAR 2147, 2222); further analyses of the Colombian populations (identified as *H*. cf. *talamacae* by Díaz-Ricaurte & Guevara-Molina, 2020) is necessary to determine if they are conspecific with *H. mashpi* sp. nov. *Hyalinobatrachium nouns* sp. nov. is sister to the clade formed by *H. mashpi* sp. nov. and the Colombian populations; genetic distances to Colombian populations are also considerable (4.7–5.1%). More distantly related taxa include two species from Central America, *H. vireovittatum* (Starrett & Savage, 1973) and *H. talamancae* (Taylor, 1952).

***Hyalinobatrachium mashpi*** new species

LSID: 0815B7E6-33FB-42D9-A367-4FB50885C256

**Suggested English name:** Mashpi Glassfrog

**Suggested Spanish name:** Rana de Cristal de Mashpi

**Holotype.** CJ11642, adult male from San Vicente River (0.16334 N, 78.86736 W; 1,040 m a.s.l.), Mashpi Reserve, Pichincha Province, Ecuador, collected by Jaime Culebras and Carlos Morochz on 28 September 2019.

**Paratopotypes.** CJ11643–44, adult males with same data as holotype.

**Paratypes.** MZUTI-3921, adult male from Amagusa River (0.15469 N, 78.85322 W; 1,137 m a.s.l.), Amagusa Reserve, Pichincha Province, Ecuador, collected by Carlos Morochz and Lucas Bustamante on 14 December 2014. CJ11645, adult male from tributary of the Mashpi River (0.11463 N, 78.88307 W; 1,126 m a.s.l.), Tayra Reserve, Pichincha Province, Ecuador, collected by Jaime Culebras on 28 October 2019.

**Figure 2:**
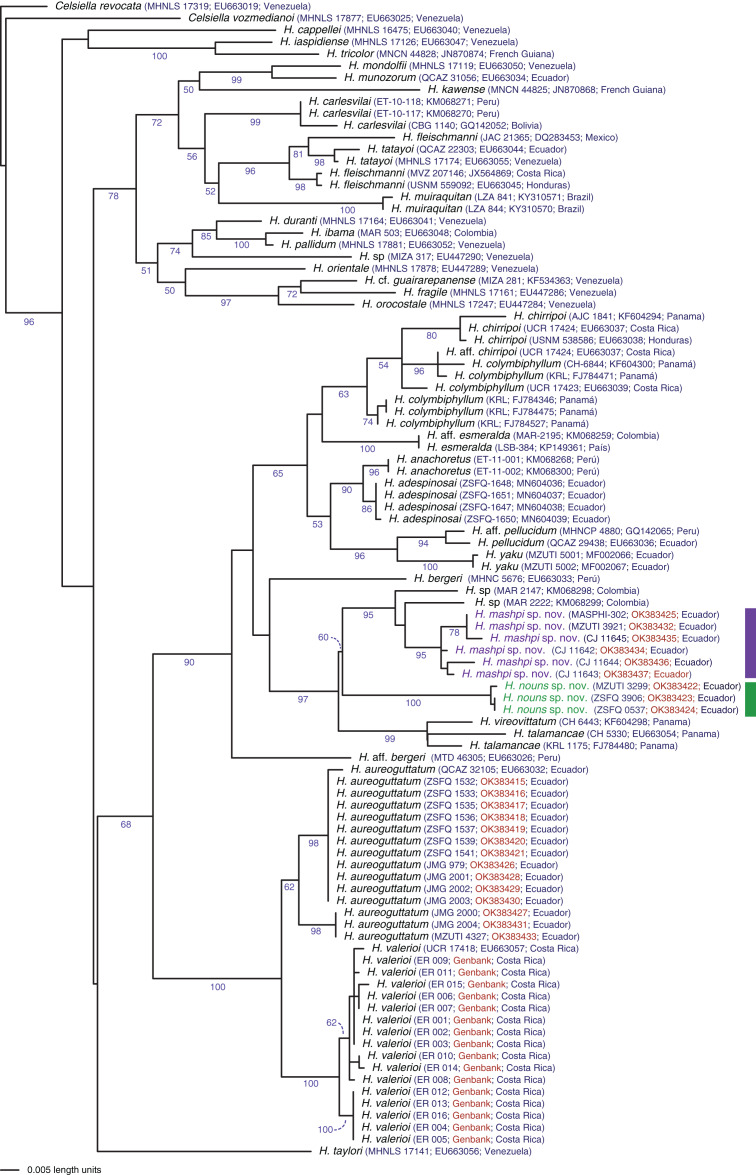
Phylogenetic position of *Hyalinobatrachium mashpi* sp. nov. and *H. nouns* sp. nov. Phylogenetic relationships of *Hyalinobatrachium* inferred from the 16S mitochondrial gene under ML criterion. All sequences were downloaded from GenBank, except those in red (Material S2). Genbank codes are listed next to each terminal. Associated locality data is available at Genbank, as well as in Guayasamin et al. (2008, 2020), Castroviejo-Fisher et al. (2014), and Twomey, Delia & Castroviejo-Fisher (2014).

**Generic placement.** The new species is placed in the genus *Hyalinobatrachium* Ruiz-Carranza & Lynch (1991), as modified by Guayasamin et al. (2009), on the basis of morphological and molecular data. The molecular phylogeny (Fig. 2) places the new species within the genus *Hyalinobatrachium* with high confidence. Phenotypically, the main diagnostic traits of *Hyalinobatrachium* are: (1) completely transparent ventral parietal peritoneum; (2) digestive tract and bulbous liver are covered by iridophores; (3) absent humeral spines; (4) white bones in life; (5) males call from the undersides of leaves; (6) females place the eggs on the undersides of leaves; (7) males provide extended parental care; and (8) tympanum with an orientation that places it almost on a horizontal plane (instead of a more lateral plane as observed in other glassfrog genera). All the aforementioned characteristics are present in *Hyalinobatrachium mashpi* sp. nov. We note that we have observed males on the same leaves as egg clutches for continuous days, but additional studies are necessary to confirm that these observations actually represent extended paternal care.

**Figure 3:**
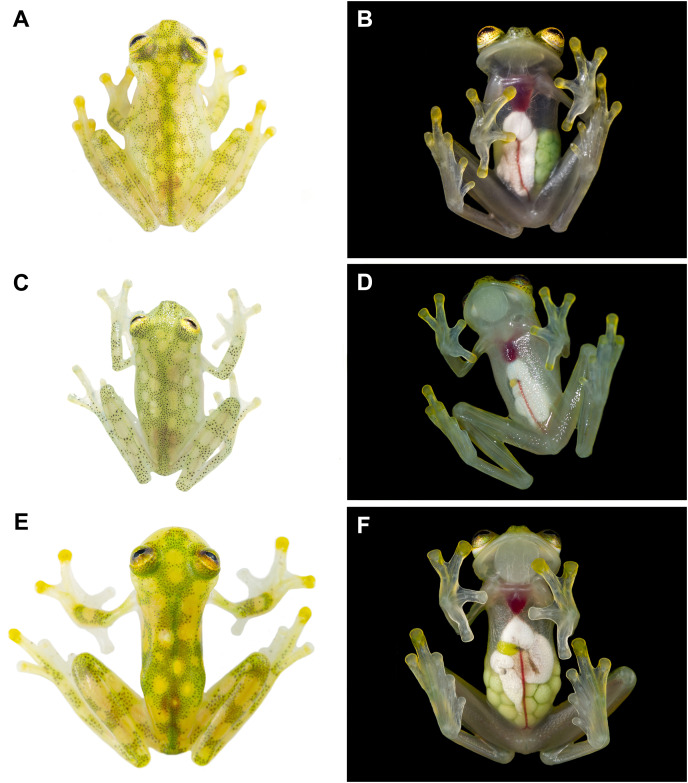
Dorsal and ventral photos of glassfrogs in life. (A) Male of *Hyalinobatrachium mashpi* sp. nov., CJ11642 (holotype). (B) Gravid female of *H. mashpi* sp. nov., Mashpi Reserve, Ecuador. (C) Male of *H. nouns* sp. nov., ZSFQ0537. (D) Male of *H*. *nouns* sp. nov., MZUTI3299 (holotype). (E) Male of *H. aureoguttatum*, Ecuador. (F) Gravid female of *H. aureoguttatum*, Ecuador. *Photos by Jaime Culebras (A, B, D, E, F) and Ross Maynard (C)*.

**Figure 4:**
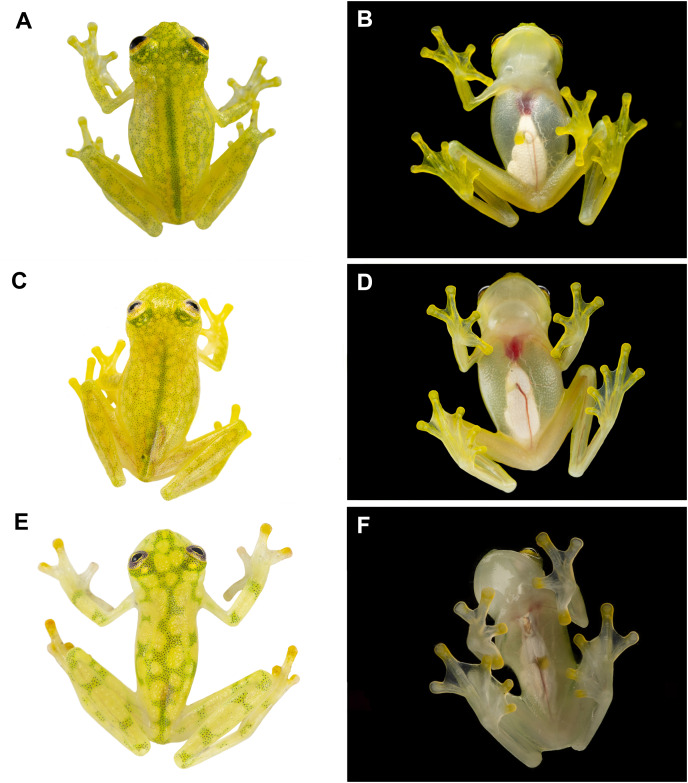
Dorsal and ventral photos of glassfrogs in life. (A, B) Male of *Hyalinobatrachium vireovittatum*, Costa Rica. (C, D) Male of *H. talamancae*, Costa Rica. (E, F) Male of *H. valerioi*, Costa Rica. *Photos by Jaime Culebras (A, C, D, E, F) and Josué Alberto Vargas (B)*.

**Figure 5:**
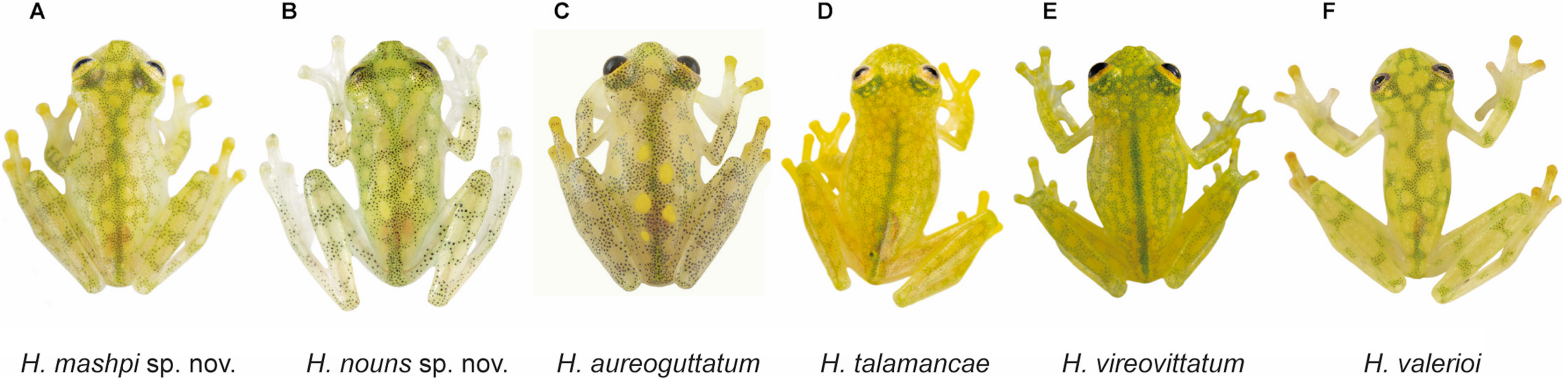
Dorsal patterns of glassfrogs in life. (A) *Hyalinobatrachium mashpi* sp. nov., CJ11642 (holotype). (B) *H. nouns* sp. nov., ZSFQ0537. (C) *H*. *aureoguttatum*, SC 435. (D) *H. talamancae*, Costa Rica. (E) *H*. *vireovittatum*, Costa Rica. (F) *H. valerioi*, Costa Rica.*Photos by Jaime Culebras (A, D, E, F), Jose Vieira (B) and Luis Coloma (C)*.

**Diagnosis.** *Hyalinobatrachium mashpi* sp. nov. is distinguished from other species in the genus mainly by its dorsal coloration (*i.e*., head with light yellow spots that may form an interorbital bar; dorsum lime green with small light yellow spots) and by its transparent pericardium (*i.e*., red heart visible in ventral view). *Hyalinobatrachium mashpi* sp. nov. is most similar to *H. aureoguttatum, H. talamancae*, *H. valerioi, H. vireovittatum*, and the new species described below. Differences among these species are indicated in Table 1 and Figs. 3–5. The new species is morphologically cryptic with *Hyalinobatrachium nouns* sp. nov. (described below); however, the two new species exhibit a considerable genetic distance (16S; 4.6–4.7%), which is particularly remarkable given that they are found at relatively close geographic proximity (straight distance = 18.9 km), but separated by the Intag-Guayllabamba river valley.

**Table 1:** Differences between *Hyalinobatrachium mashpi* sp. nov., *H. nouns* sp. nov., and similar and closely related species.

Species	SVL in mm (adult males)	Dorsal pattern	Hear coloration in life	Interorbital bar	Type of call	Biogeographic distribution/Country/Elevation (m a.s.l.)	Sources
*H. adespinosai*	20.5–22.2	Pale yellowish green with small pale yellow spots and minute gray to black melanophores	Red	Absent	Pulsed	Amazonian slopes of the Andes Ecuador 1,670–1,795	Guayasamin et al. (2019a)
*H. anachoretus*	20.6–21.4	Apple green with small yellow spots and minute melanophores	Red	Absent	Pulsed	Amazonian slopes of the Andes Peru 2,001–2,050	Twomey, Delia & Castroviejo-Fisher (2014)
*H. aureoguttatum*	20.4–24.0	Greenish yellow with large, bright yellow spots and, in some populations, dark flecks	Usually red, but also red and white, or white	Usually absent	Tonal	Chocó, Pacific slopes of Andes Colombia, Ecuador, Panama 0–1,340	Barrera-Rodriguez & Ruiz-Carranza (1989), Guayasamin et al. (2020), this study
*H. bergeri*	20.3–22.4	Apple green with small yellow spots and minute melanophores	Mostly white	Absent	Tonal	Amazonian lowlands and Amazonian slopes of the Andes Peru, Bolivia 300–1,980	Castroviejo-Fisher et al. (2009)
*H. chirripoi*	24–27	Greenish yellow with small yellow spots	Red	Absent	Pulsed	Central America (Costa Rica, Panama); Chocó (Colombia and Ecuador) 0–320	Savage (2002), Kubicki (2007), Guayasamin et al. (2020)
*H. colymbiphyllum*	23–30	Greenish yellow with yellow spots	Red	Absent	Pulsed	Central America (Costa Rica, Honduras, Panama); Chocó (Colombia) 0–1,710	McCranie & Wilson (2002), Savage (2002), Kubicki (2007)
*H. esmeralda*	18.4–22.3	Greenish yellow with yellow spots and some minute dark dots	Red or red and white	Absent	Unknown	Amazonian slopes of the Andes Colombia 1,026–1,700	Ruiz Carranza & Lynch (1998), Acosta-Galvis (2017), Twomey, Delia & Castroviejo-Fisher (2014)
***H. mashpi* sp. nov.**	19.7–20.9	Greenish yellow with small and diffuse yellow spots	Red	Usually present	Pulsed	Pacific slopes of the Andes Ecuador 976–1,137	This study
***H. nouns* sp. nov.**	19.1–21.3	Greenish yellow with small and diffuse yellow spots	Red	Usually present	Unknown	Pacific slopes of the Andes Ecuador 1,177–1,420	This study
*H. pellucidum*	20.4–21.4	Greenish yellow with small yellow spots	White	Absent	Tonal	Amazonian slopes of the Andes Ecuador, Peru 523–1,740	Lynch & Duellman (1973), Guayasamin et al. (2020)
*H. valerioi*	19.5–24.0	Greenish yellow with large and diffuse yellow spots	Red or red and white	Usually absent	Tonal	Central América, Chocó, Pacific slopes of the Andes Costa Rica, Colombia, Ecuador 0–1,500	Savage (2002), Kubicki (2007), Guayasamin et al. (2020)
*H. vireovittatum*	21.5–23.0	Greenish yellow with small yellow spots. Dark green middorsal stripe outlined by yellow paravertebral stripes	Red	Present or absent	Tonal	Central America Costa Rica 250–1,957	Savage (2002), Kubicki (2007), Campos-Villalobos et al. (2020)
*H. talamancae*	24–26	Greenish yellow with small yellow spots. Dark green middorsal stripe present.	Red	Present or absent	Tonal	Central America Costa Rica 475–1,600	Kubicki (2006, 2007); Zamora-Roda, Herrera-Martínez & Salazar (2021)
*H. yaku*	20.8–22.3	Green with small yellow spots and minute melanophores; posterior head and anterior half of the body with few small dark green spots placed middorsally	Red	Absent	Tonal	Amazonian lowlands Ecuador 300–360	Guayasamin et al. (2017a)

**Note:**

Sources of traits are indicated in the last column.

**Characterization.** The following combination of characters are found in *Hyalinobatrachium mashpi* sp. nov.: (1) dentigerous process of the vomer lacking teeth; (2) snout truncate in dorsal view and slightly protruding in lateral view; (3) tympanum oriented almost horizontally; tympanic annulus barely visible, hidden under skin; tympanic membrane differentiated, with coloration similar to that of surrounding skin; (4) dorsal skin shagreen; (5) ventral skin areolate; cloacal ornamentation absent, paired round tubercles below vent absent; (6) parietal peritoneum and pericardium translucent (in life, red heart visible in ventral view); liver, viscera and testes covered by iridophores; (7) liver bulbous; (8) humeral spines absent; (9) hand webbing formula: I (2^+^–2^1/2^)—(3^–^–3) II (2–2^–^)—(3^+^–3^1/4^) III (2–2^+^)—(1^3/4^–2) IV; (10) foot webbing moderate; webbing formula: I 1^+^—(2–2^+^) II (1–1^1/3^)—2^1/4^ III (1^1/3^–1^1/2^) —(2^+^–2^1/4^) IV (2^1/2^–2^1/3^)—1 V; (11) fingers and toes with thin lateral fringes; ulnar and tarsal folds absent; (12) nuptial excrescence present as a small pad on Finger I (Type V), prepollex not enlarged; prepollical spine not projecting (spine not exposed); (13) when appressed, Finger I longer than II; (14) diameter of eye about 2 times wider than disc on Finger III; (15) coloration in life: dorsal surfaces lime green with small light yellow spots; (16) coloration in preservative: dorsal surfaces creamish white, with minute lavender melanophores; (17) eye coloration in life: iris yellow to golden-yellow; pupil surrounded by lavender ring; (18) melanophores absent from fingers and toes, except Toes IV and V; (19) males call from underside of leaves; advertisement call consisting of single pulsed note, with duration of 0.37–0.46 s, peak frequency at 5.25–5.60 kHz, maximum frequency at 5.46–5.81 kHz, and minimum frequency at 4.62–4.92 kHz; (20) males attend egg clutches located on the underside of leaves overhanging streams; clutch size of 31 or 32 embryos (*n* = 2); (21) SVL in adult males 19.7–20.9 mm (mean = 20.5; *n* = 5); females unknown; and (22) enameled tubercles absent from sides of head.

**Description of the holotype**. CJ11642, adult male with SVL 20.6 mm. Head wider than long (head width 39% of SVL; head length 78% of head width). Snout truncate in dorsal view and slightly protruding in lateral view. Loreal region concave, nostrils slightly protuberant, elliptical; internarial region concave; canthus rostralis not well defined. Eyes small, directed anterolaterally, eyes about 50° relative to midline (where anterior-facing eyes would be 90° relative to midline). Tympanum barely visible, oriented almost horizontally; tympanic membrane differentiated and pigmented as surrounding skin. Dentigerous processes on vomers absent; choanae large, oval, separated widely (distance about the same as between nostrils); tongue round, white in preservative, anterior 4/5 attached to mouth; posterior border of tongue widely notched; vocal slits present, extending along floor of mouth lateral to tongue; enameled glands absent from lower part of upper jaw. Ulnar fold absent; humeral spine absent. Relative length of fingers: II < I < IV < III; finger discs rounded, about the same size as discs on toes, disc on Finger III 42% of eye width; hand webbing reduced between Fingers I–III, moderate between Fingers III and IV, with formula I 2^+^—3^–^ II 2^–^—3^1/5^ III 2^+^—1^3/4^ IV. Prepollex concealed; subarticular tubercles round, faint; few small supernumerary tubercles present, palmar tubercle round, of moderate size and difficult to see, thenar tubercle ovoid; nuptial excrescences present as a small pad on external edge of Finger I (Type V). Hind limbs slender, tibia length 55% of SVL; tarsal fold absent; discs of toes round; inner metatarsal tubercle small, outer metatarsal tubercle round, both very difficult to distinguish. Webbing formula of feet: I 1^+^—2 II 1—2^1/4^ III 1^1/2^—2^+^ IV 2^1/2^—1 V. In preservative, dorsal skin creamish white, with minute dark lavender melanophores (only visible under the stereomicroscope); dorsal skin shagreen; skin on venter areolate; cloacal opening at level of upper thighs, small and non-enameled cloacal warts present. Parietal peritoneum and pericardium translucent (in life, the red heart is visible ventrally); urinary bladder lacking iridophores; liver, viscera, and tested fully covered by iridophores. Kidneys rounded, approximately bean-shaped; liver bulbous.

**Coloration in life.** Dorsal surfaces apple green to yellowish green with diffuse yellow spots; head with light yellow spots that may form an interorbital bar. Melanophores absent from fingers and toes, except Toes IV and V. Ventrally, parietal peritoneum and pericardium transparent, with a red heart always visible. Gall bladder and urinary bladder covered by translucent peritonea; hepatic and visceral peritonea covered by white iridophores; ventral vein red. Iris yellow, with numerous minute lavender spots. Bones white.

**Coloration in preservative**. Dorsal surfaces creamish white dotted with minute dark lavender melanophores; venter uniform cream, with partial translucence; pericardium translucent; visceral peritoneum covered by iridophores. Iris white with minute lavender melanophores. Melanophores absent from hands and feet, except from some few present on dorsal surfaces of Toes IV and V.

**Measurements of holotype (in mm)**. CJ11642, adult male. SVL = 20.6, femur length = 11.4, tibia length = 11.3, foot length = 9.6, head length = 6.2, head width = 8.0, interorbital distance = 2.4,eye diameter = 2.6, tympanum diameter = 0.6, arm length = 4.2, hand length = 6.3, Finger I length = 4.6, Finger II length = 4.1, width of Finger III = 1.1.

**Figure 6:**
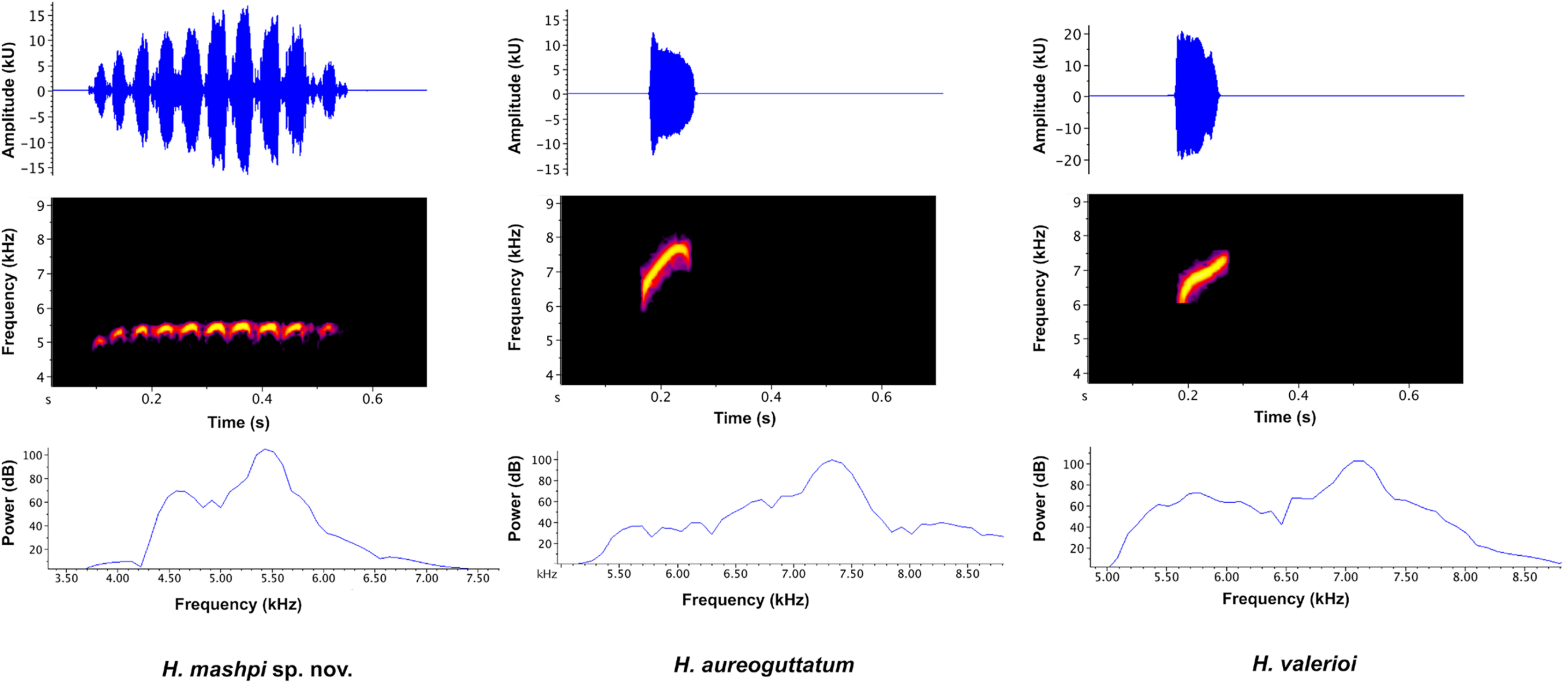
Visual representation of *Hyalinobatrachium mashpi* sp. nov. advertisement call, with comparisons of two similar species, *H. aureoguttatum* and *H. valerioi*. The call of each species is depicted in three forms: (Top) oscillograms, waveforms representing amplitude changes over time; (Middle) spectrograms, plots of frequency over time, with higher amplitudes represented by brighter colors; and (Bottom) power spectra, representing the relative amplitude of each frequency.

**Figure 7:**
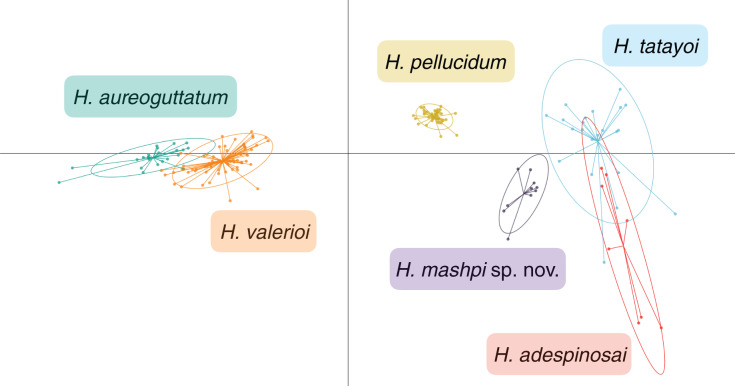
Discriminant analysis of principal components (DAPC) of glassfrog species’ calls. Variables analyzed include: peak frequency, maximum frequency, minimum frequency, call duration, and inter-call duration. Sample size as follows: *H. adespinosai* one individual, 10 calls; *H. aureoguttatum* six individuals, 24 calls; *H. mashpi* sp. nov. two individuals, 12 calls; *H. pellucidum* one individual, 41 calls; *H. tatayoi* four individuals, 26 calls; *H. valerioi* three individuals, 70 calls.

**Vocalizations** (Figs. 6, 7). We measured call variables from two individuals, each from a different locality, Mashpi Reserve (CJ11642; call code LBE-C-051) and Tayra Reserve (CJ11645; call code LBE-C-052). The call of *Hyalinobatrachium mashpi* sp. nov. (Fig. 6) consists of a single pulsed (amplitude-modulated) note, which starts with one lower-frequency pulse followed by ~9 more consistent pulses at a slightly higher dominant frequency. We analyzed variables from both individuals: four calls from CJ11645 and eight calls from CJ11642. Calls in our field recordings had a duration of 0.373–0.461 s (mean = 0.425 ± 0.027 SD, *n* = 12). Time between calls ranged from 10.07–17.48 s (mean = 12.80 ± 2.166 SD, *n* = 10); intervals between *H. mashpi* calls were longer when a sympatric glassfrog (*Espadarana prosoblepon*) called in the interim period. Peak frequency was 5.25–5.6 kHz (mean = 5.38 kHz ± 0.12 SD; *n* = 12), with a maximum frequency of 5.46–5.81 kHz (mean = 5.38 kHz ± 0.11 SD; *n* = 12) and a minimum frequency of 4.62–4.92 kHz (mean = 4.79 kHz ± 0.10 SD; *n* = 12).

We compared the calls of *H. mashpi* sp. nov. to those of phenotypically and/or genotypically similar species within the same genus: *H. adespinosai, H. aureoguttatum*, *H. pellucidum*, *H. tatayoi*, and *H. valerioi* (Table 2). The call of *H. adespinosai* is a single pulsed (amplitude-modulated) note, consisting of ~12 pulses (mean call duration = 0.54 s ± 0.007 SD, *n* = 10). Time between calls ranged from 10.87–30.04 s (mean inter-call interval = 20.12 s ± 8.77 SD). Mean peak/fundamental frequency was 4.94 kHz (±0.07 SD; range = 4.87–5.04 kHz), with a mean maximum frequency of 5.11 (±0.08 SD; range = 5.0–5.25 kHz) and a mean minimum frequency of 4.57 (±0.15 SD; range = 4.32–4.75 kHz).

**Table 2:** Acoustic differences between *Hyalinobatrachium mashpi* sp. nov. and related species.

Species	Call codes	Call structure	Call duration (s)	Inter-call interval (s)	Peak frequency (kHz)	Maximum frequency (kHz)	Minimum frequency (kHz)
***H. mashpi*** **sp. nov.** two individuals, 12 calls	LBE-C-051, LBE-C-052	1 note per call; pulsed; 8–10 pulses per note	0.37–0.46 (0.43 ± 0.03)	10.07–17.48 (12.8 ± 2.17)	5.25–5.6 (5.38 ± 0.12)	5.46–5.81 (5.62 ± 0.11)	4.62–4.92 (4.79 ± 0.10)
***H. adespinosai*** one individual 10 calls	LBE-C-050	1 note per call; pulsed; ~12 pulses per note	0.448–0.646 (0.543 ± 0.07)	10.87–30.04 (20.12 ± 8.77)	4.87–5.04 (4.94 ± 0.07)	5.0–5.25 (5.11 ± 0.08)	4.32–4.75 (4.57 ± 0.15)
***H. aureoguttatum*** six individuals 24 calls	LBE-C-053–057	1 note per call; tonal; harmonics present	0.078–0.087 (0.082 ± 0.002)	1.99–5.20 (3.24 ± 0.79)	6.63–7.41 (6.87 ± 0.25)	7.78–8.90 (8.11 ± 0.27)	5.16–5.91 (5.62 ± 0.21)
***H. chirripoi*** one individual two calls	LBE-C-010	1 note per call; pulsed; 12–13 pulses per note	0.235–0.274 (0.255 ± 0.03)	84.3 (only 2 notes in recording)	4.48 (4.48 ± 0)	4.99–5.77 (5.38 ± 0.05)	4.16–4.21 (4.19 ± 0.04)
***H. pellucidum*** 1 individual 41 calls	LBE-C-003	1 note per call; tonal	0.1–0.146 (0.129 ± 0.009)	1.67–5.35 (2.94 ± 0.79)	5.60–5.86 (5.70 ± 0.06)	5.86–6.14 (6.0 ± 0.06)	5.05–5.32 (5.16 ± 0.07)
***H. tatayoi*** four individuals 26 calls	LBE-C-058	1 note per call; tonal	0.076–0.276 (0.143 ± 0.04)	2.05–21.68 (7.64 ± 4.92)	4.45–5.11 (4.82 ± 1.77)	4.83–5.40 (5.14 ± 0.17)	3.30–4.61 (4.24 ± 0.34)
***H. valerioi*** three individuals 70 calls	ML201469, ML201473, ML201475	1 note per call; tonal; harmonics present	0.065–0.10 (0.079 ± 0.01)	1.76–8.00 (4.27 ± 1.21)	6.46–7.24 (6.77 ± 0.19)	7.22–7.90 (7.53 ± 0.17)	4.09–5.88 (5.12 ± 0.51)

**Note:**

For each variable, data range is followed by the mean and standard deviation in parentheses.

The call of *H. aureoguttatum* (Fig. 6; Table 2) consists of a very short, single tonal note (mean call duration = 0.082 s ± 0.002 SD, *n* = 24). Time between calls ranged from 1.99–5.20 s (mean inter-call interval = 3.24 s ± 0.79 SD, *n* = 23). Mean peak/fundamental frequency was 6.86 kHz (±0.25 SD; range = 6.55–7.41 kHz; *n* = 24 calls). Two harmonics are present. We measured call variables from individuals recorded in Canandé (0.5112 N, 79.1343 W; 457 m), Esmeraldas Province, Ecuador, in December 2018 by AVA (LBE-053–55), and in Mashpi Lodge Reserve (0.17057 N, 78.888 W; 721–723 m) in March 2019 by RMB (LBE-056, 057).

The call of *H. chirripoi* is a single pulsed (amplitude-modulated) note, consisting of ~12 pulses (mean call duration = 0.255 s ± 0.03 SD, *n* = 2). Since our recording only included two bouts of calling, we were unable to include *H. chirripoi* in the DAPC analysis. The interval between the two calls was 84.3 s. Peak/fundamental frequency was 4.48 kHz, with a maximum frequency of 4.99–5.77 kHz and a minimum frequency of 4.16–4.21 kHz. We measured call variables from one individual recorded in Reserva Itapoa (0.51307 N, 79.134 W; 321 m), Esmeraldas Province, Ecuador, in July 2016 by JC (LBE-019).

The call of *H. pellucidum* consists of a short, single tonal note (mean call duration = 0.129 s ± 0.009 SD, *n* = 41). Time between calls ranged from 1.67–5.35 s (mean inter-call interval = 2.94 s ± 0.79 SD). Mean peak/fundamental frequency was 5.70 kHz (±0.06 SD; range = 5.60–5.86 kHz), with a mean maximum frequency of 6.0 (± 0.06 SD, range 5.86–6.14 kHz) and a mean minimum frequency of 5.16 (±0.07 SD, range 5.05–5.32 kHz). We measured call variables from one individual (USNM 286708) recorded at Río Azuela, Napo Province, Ecuador, by Roy McDiarmid on February 23th, 1979.

The call of *H. tatayoi* consists of a short, single tonal note (mean call duration = 0.143 s ± 0.04 SD, *n* = 26). Time between calls ranged from 2.05–21.68 s (mean inter-call interval = 7.64 s ± 4.92 SD). Mean peak/fundamental frequency was 4.82 kHz (±1.77 SD; range = 4.45–5.11 kHz), with a mean maximum frequency of 5.14 (±0.17 SD, range 4.83–5.40 kHz) and a mean minimum frequency of 4.24 (±0.34 SD, range 3.30–4.61 kHz). We measured call variables from four individuals recorded in Jama Coaque Reserve (0.108264 S, 80.117701 W; 700 m), Manabí Province, Ecuador, in March 2019 by RMB.

The call of *H. valerioi* (Fig. 6) consists of a single tonal note (mean call duration = 0.079 s ± 0.01 SD, *n* = 70). Time between calls ranged from 1.76–8.00 s (mean inter-call interval = 4.27 s ± 1.2 SD). Mean peak frequency was 6.77 kHz (±0.19 SD; range = 6.46–7.24 kHz). Harmonics are likely present but are difficult to discern in the available recordings. We measured call variables from three individuals recorded in Costa Rica (Limón and Rincón de Oso) by Roy McDiarmid. We used the following recordings from the Macaulay Library at the Cornell Lab of Ornithology: ML212787, ML212788, and ML213430.

Results from the discriminant analysis of principal components (DAPC) revealed that the calls of *H. mashpi* sp. nov. cluster separately, and are thus acoustically distinct from *H. adespinosai, H. aureoguttatum*, *H. pellucidum*, *H. tatayoi*, and *H. valerioi* (Fig. 7). Overlap occurred between *H. aureoguttatum* and *H. valerioi* clusters, as well as between *H. tatayoi* and *H. adespinosai* clusters. This suggests that the calls of these pairs may not be adequate for species identification alone; more field recordings with genetic verification of the calling species are thus recommended for future studies. Nearly all (99.9%) of the variance was retained by three principal components. Table 3 lists the eigenvalues and variable loadings of each principal component.

**Table 3:** Results from the discriminant analysis of principle components (DAPC), comparing the advertisement calls of *Hyalinobatrachium mashpi* sp. nov. with those of closely related species (see Fig. 7).

	PC1	PC2	PC3
**PCA** **Eigenvalues**	23.76	1.48	0.16
**PCA Loadings**			
***Peak Frequency***	0.08	−0.57	−0.09
***Call (Note) Duration***	−0.02	0.023	0.05
***Inter-call Interval***	−0.99	−0.14	0.02
***Maximum Frequency***	0.11	−0.78	−0.19
***Minimum Frequency***	0.04	−0.21	0.98

**Note:**

Most variation (99.9%) was retained by three principal components.

**Figure 8:**
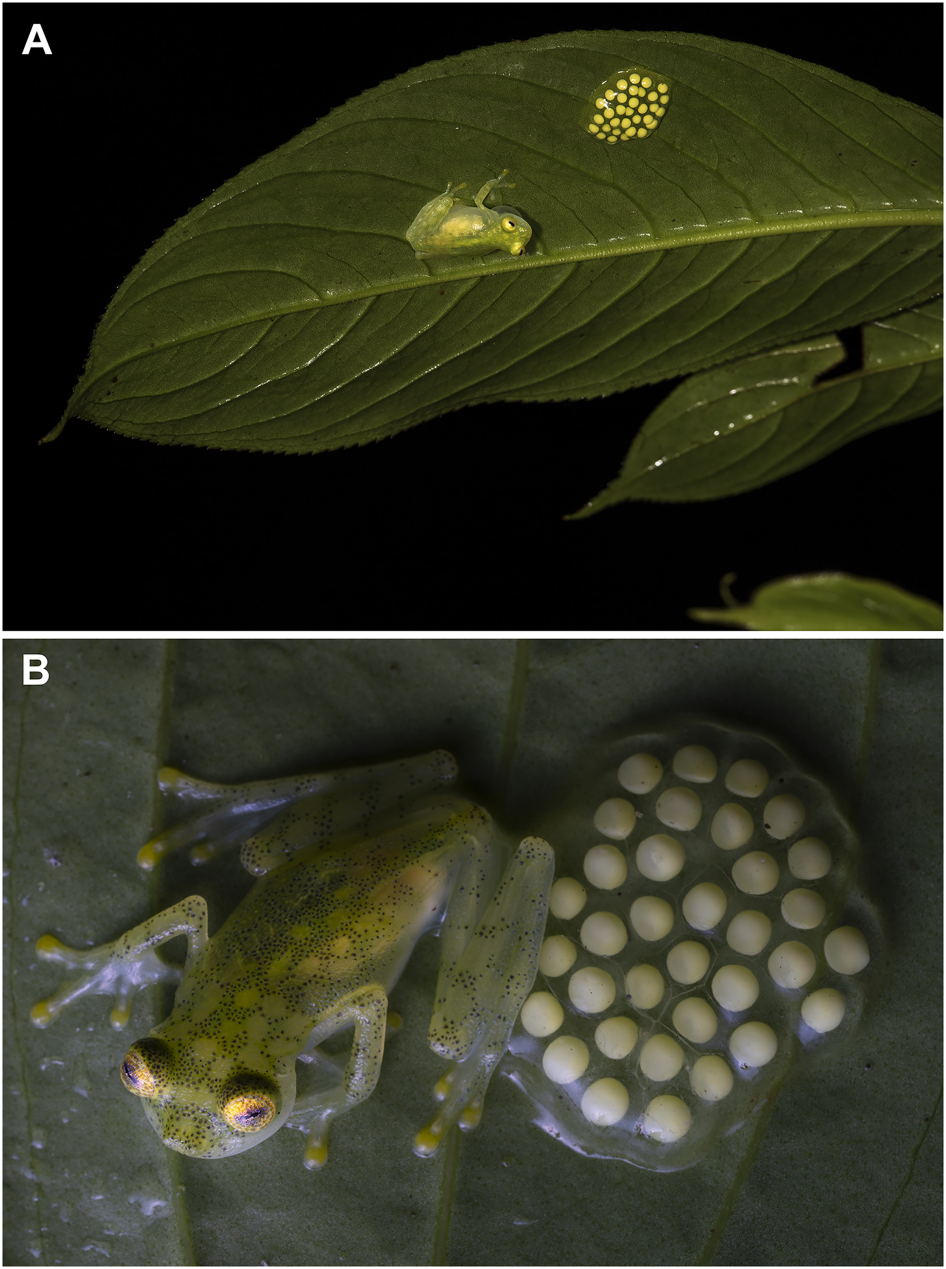
Parental care in *Hyalinobatrachium mashpi* sp. nov. (A) Male calling at San Vicente River, Mashpi Reserve, Pichincha Province, Ecuador. (B) Male at tributary of the Mashpi River, Tayra Reserve, Pichincha Province, Ecuador. Photos by Carlos Morochz (A) and Jaime Culebras (B).

**Figure 9:**
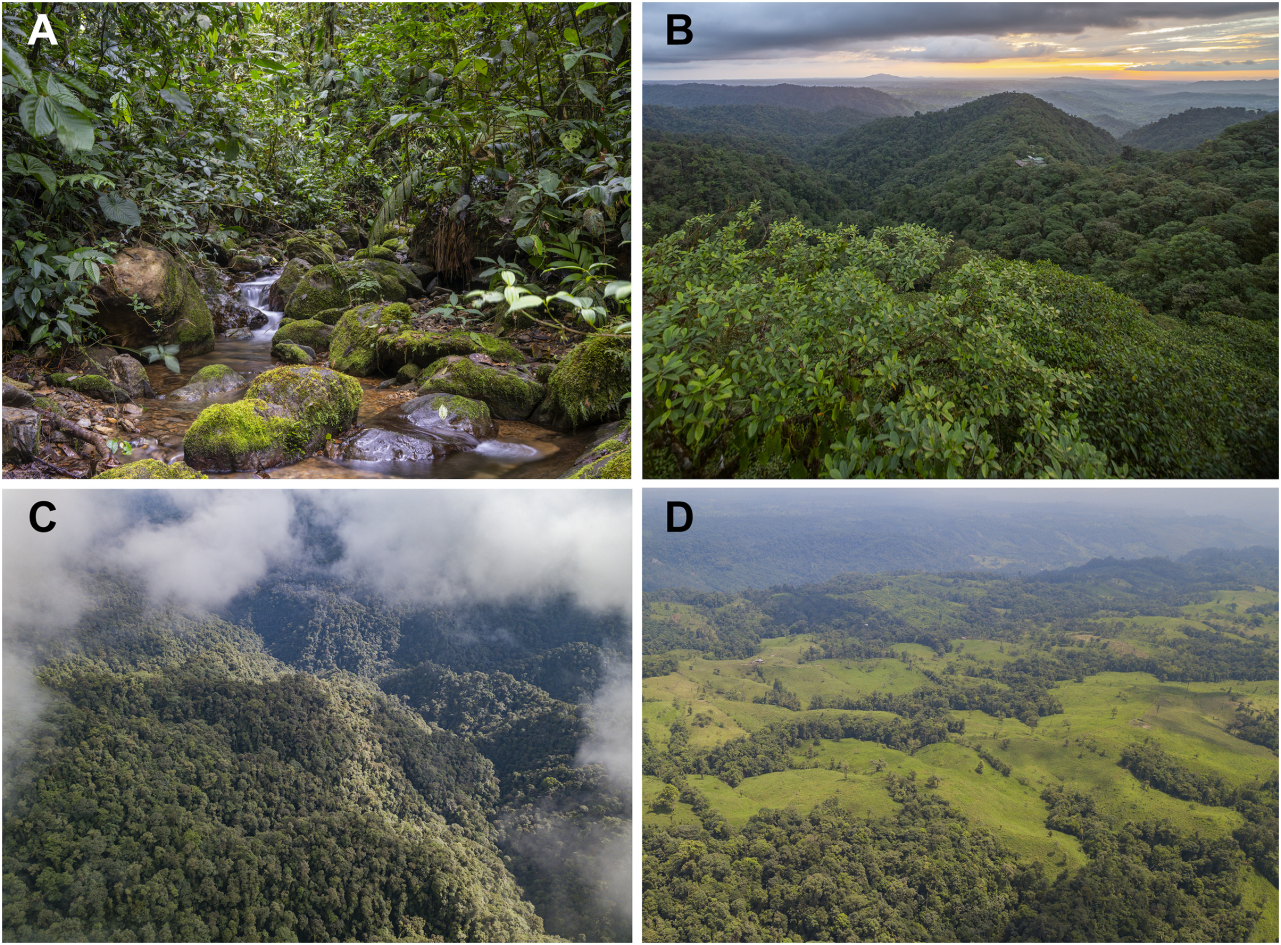
Habitat of *Hyalinobatrachium mashpi* sp. nov. (A) Tributary of the Mashpi River, Tayra Reserve, Pichincha Province, Ecuador. (B) Mashpi Reserve, Pichincha Province, Ecuador. (C) Tayra Reserve, Pichincha Province, Ecuador. (D) Habitat loss in the vicinity of Tayra Reserve, Pichincha Province, Ecuador. Photos by Jaime Culebras.

**Natural history.** Most individuals of *Hyalinobatrachium mashpi* sp. nov. were found on the underside of leaves among riverine vegetation (Figs. 8, 9). These frogs are difficult to observe because they are found 3–14 m above ground along steep creeks. Males have been observed calling in the months of April, May, June, August, September, October and November. Males that were guarding egg clutches while calling were observed during the rainy season (18 February 2019; 7 May 2021) and dry season (October 2014, June 2015, and August 2021). Examined egg clutches contain 31–34 eggs (*n* = 3). A female with mature eggs visible through the skin was observed on 27 May 27 2015.

**Figure 10:**
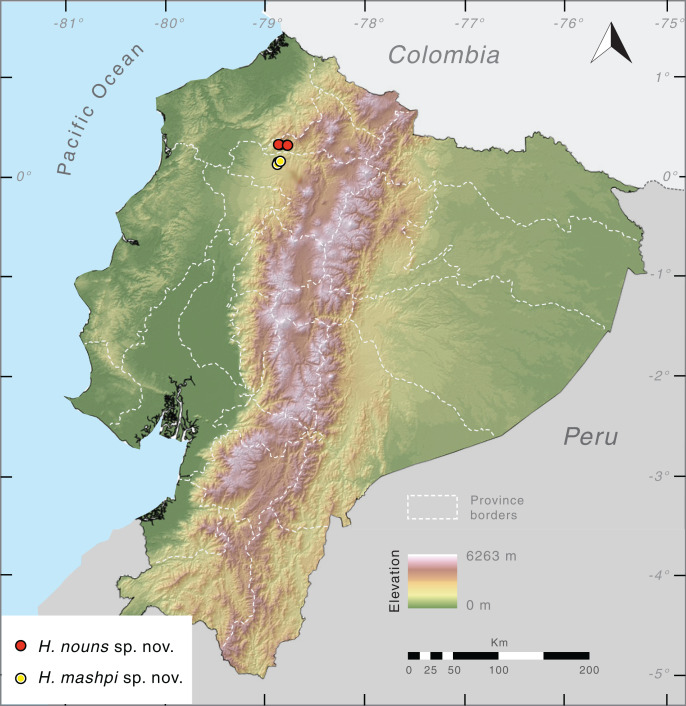
Distribution of *Hyalinobatrachium mashpi* sp. nov. and *H. nouns* sp. nov. in Ecuador. Note that localities of the two new taxa are separated by the Intag-Guayllabamba valley.

**Distribution** (Fig. 10). *Hyalinobatrachium mashpi* sp. nov. is only known from the following localities in the Mashpi river basin, Pichincha Province, Ecuador: (i) Mashpi Lodge Reserve (San Vicente River, 1,040–1,101 m; Laguna River, 1,069 m); (ii) Amagusa Reserve (Amagusa River, 1,137 m; Mashpi Chico River, 1,130 m); and (iii) Tayra Reserve, 976–1,126 m. Unidentified and closely related frogs from Colombia (Departamento de Risaralda, MAR 2147; Departamento de Valle del Cauca; MAR 2222; Fig. 2) may prove to be conspecifics of *H. mashpi*.

**Evolutionary relationships**. Our phylogenetic analyses (Fig. 2) reveal *Hyalinobatrachium mashpi* sp. nov. as sister to undetermined haplotypes from the Colombian Andes (MAR 2147, 2222) and a new species from the Toisán Mountain Range, described below. Other closely related taxa are endemic to Central America: *H. vireovittatum* and *H. talamancae* (Fig. 2).

**Etymology.** The specific epithet *mashpi* is used as a noun in apposition and refers to the Mashpi area in northwestern Ecuador. There are several conservation efforts to preserve the last patches of forest remaining in Mashpi (*e.g*., Mashpi Lodge Reserve, Tayra Reserve, Amagusa Reserve, Mancomunidad del Chocó Andino, Chocó Andino Biosphere Reserve). *Mashpi* is a Yumbo word that means ‘friend of water’, an apt description of this glassfrog, which depends on healthy streams for its reproduction.

**Conservation status.** We recommend that *Hyalinobatrachium mashpi* be listed as Endangered, following IUCN Red List criteria B1ab(iii): extent of occurrence estimated to be less than 5,000 km^2^; known to exist at no more than 5 localities; and continuing decline, observed, inferred or projected, in area, extent, and/or quality of habitat. The main threats for this species are habitat loss and contamination due to cattle ranching, agriculture, and mining activities (see Discussion).

***Hyalinobatrachium nouns*** new species

LSID: 1A908651-9A82-4DCA-9960-E8DC525F5ADF

**Suggested English name:** Nouns’ Glassfrog

**Suggested Spanish name:** Rana de Cristal de Nouns

**Holotype.** MZUTI 3299, adult male from stream in Bosque Protector Los Cedros (0.310 N, 78.781 W; 1,420 m a.s.l.), Cordillera de Toisán, Imbabura Province, Ecuador, collected by Mariela Palacios, Jaime Culebras and Juan M. Guayasamin, on 12 March 2012.

**Paratypes.** CJ7703, adult male from stream in Bosque Protector Los Cedros (0.30241 N, 78.78558 W; 1,229 m a.s.l.), Cordillera de Toisán, Imbabura Province, Ecuador, collected by Morley Read and Arturo Guasti on 8 November 2017. CJ7722, adult male from stream in Bosque Protector Los Cedros (0.30191 N, 78.78513 W; 1,241 m a.s.l.), Cordillera de Toisán, Imbabura province, Ecuador, collected by Morley Read and Arturo Guasti on November 11^th^ 2017. CJ7723, adult male from stream in Bosque Protector Los Cedros (0.30302 N, 78.78674 W; 1,313 m a.s.l.), Cordillera de Toisán, Imbabura province, Ecuador, collected by Morley Read and Arturo Guasti on November 11^th^ 2017. ZSFQ-0537, adult male from stream in Río Manduriacu Reserve (0.31126 N, 78.8588 W; 1,254 m a.s.l.), Cordillera de Toisán, Imbabura province, Ecuador, collected by José Vieira, Scott Trageser, and Ross J. Maynard on 10 February 2018. ZSFQ-3906, metamorph from stream in Río Manduriacu Reserve (0.3099 N, 78.8567 W; 1,202 m a.s.l.), Cordillera de Toisán, Imbabura province, Ecuador, collected by Ross J. Maynard and Jaime Culebras on 23 November 2019.

**Generic placement.** Based of morphological and molecular data, the new species is placed in the genus *Hyalinobatrachium sensu* Ruiz-Carranza & Lynch, as modified by Guayasamin et al. (2009). The molecular phylogeny (Fig. 2) places the new species within the genus *Hyalinobatrachium* with high confidence. Phenotypically, *Hyalinobatrachium nouns* sp. nov. shares the following diagnostic traits of the genus *Hyalinobatrachium*: (1) completely transparent ventral parietal peritoneum; (2) digestive tract and bulbous liver are covered by iridophores; (3) absent humeral spines; (4) white bones in life; (5) males call from the undersides of leaves, (6) females place the eggs on the undersides of leaves; (7) males provide extended parental care; and (8) tympanum with an orientation that places it almost on a horizontal plane (instead of a more lateral plane as observed in other glassfrog genera). All the aforementioned characteristics are present in *Hyalinobatrachium nouns* sp. nov. We note that we have observed males on the same leaves as egg clutches for consecutive days, suggesting the possibility of parental care, but additional studies are necessary to confirm that these observations actually represent extended paternal care as observed in other *Hyalinobatrachium* species (see Delia, Bravo-Valencia & Warkentin, 2017).

**Diagnosis.** *Hyalinobatrachium nouns* sp. nov. is distinguished from other species in the genus mainly by its dorsal coloration (*i.e*., head with light yellow spots that may form an interorbital bar; dorsum lime green with small light yellow spots) and by its transparent pericardium. *Hyalinobatrachium nouns* sp. nov. is most similar to *H. aureoguttatum, H. mashpi* sp. nov., *H. talamancae*, *H. valerioi*, and *H. vireovittatum*. Differences among these species are indicated in Table 1 and Figs. 2–4. The new species is morphologically cryptic with *Hyalinobatrachium mashpi* sp. nov. (described above), but they exhibit a considerable genetic distance (16S; 4.6–4.7%), which is remarkable given that they are found at relatively close geographic proximity (straight distance = 18.9 km), but separated by the Intag-Guayllabamba river valley.

**Characterization.** The following combination of characters are found in *Hyalinobatrachium nouns* sp. nov.: (1) dentigerous process of the vomer lacking teeth; (2) snout truncate in dorsal view and slightly protruding in lateral view; (3) tympanum oriented almost horizontally; tympanic annulus barely visible, hidden under skin; tympanic membrane differentiated, with coloration similar to that of surrounding skin; (4) dorsal skin shagreen; (5) ventral skin areolate; cloacal ornamentation absent, paired round tubercles below vent absent; (6) parietal peritoneum and pericardium translucent (in life, red heart visible in ventral view); liver, viscera and testes covered by iridophores; (7) liver bulbous; (8) humeral spines absent; (9) hand webbing formula: I (2^+^–2)—(2–2^1/2^) II (1^+^–1^1/2^)—(3–3^+^) III (2–2^+^)—(1^1/2^–1^3/4^) IV; (10) foot webbing moderate; webbing formula: I (1–1^+^)—(2^–^–2) II (1–1^+^)—(2^+^–2^1/2^) III 1—(2^+^–2^1/3^) IV (2^1/4^–2^1/3^)—(1^+^–1^1/3^) V; (11) fingers and toes with thin lateral fringes; ulnar and tarsal folds absent; (12) nuptial excrescence present as a small pad on Finger I (Type V), prepollex not enlarged; prepollical spine not projecting (spine not exposed); (13) when appressed, Finger I longer than II; (14) diameter of eye about 2 times wider than disc on Finger III; (15) coloration in life: dorsal surfaces lime green with small light yellow spots; (16) coloration in preservative: dorsal surfaces creamish white, with minute lavender melanophores; (17) eye coloration in life: iris yellow to golden-yellow; pupil surrounded by lavender ring; (18) melanophores absent from fingers and toes, except Toes IV and V; (19) males call from underside of leaves; advertisement call unknown; (20) parental care unknown; clutch size unknown; (21) SVL in adult males 19.1–21.3 mm (mean = 20.3; *n* = 4), females unknown; and (22) enameled tubercles absent from sides of head.

**Description of the holotype**. MZUTI 3299, adult male with SVL 19.1 mm. Head wider than long (head width 39% of SVL; head length 80% of head width). Snout truncate in dorsal view and slightly protruding in lateral view. Loreal region concave, nostrils slightly protuberant, elliptical; internarial region concave; canthus rostralis not well defined. Eyes small, directed anterolaterally, eyes about 50° relative to midline (where anterior-facing eyes would be 90° relative to midline). Tympanum visible, oriented almost horizontally; tympanic membrane differentiated and pigmented as surrounding skin. Dentigerous processes on vomers absent; choanae large, oval, separated widely (distance about the same as between nostrils); tongue round, white in preservative, anterior 4/5 attached to mouth; posterior border of tongue slightly notched; vocal slits present, extending along floor of mouth lateral to tongue; enameled glands absent from lower part of upper jaw. Ulnar fold absent; humeral spine absent. Relative length of fingers: II < I < IV < III; finger discs rounded, about the same size as discs on toes, disc on Finger III 41% of eye width; hand webbing reduced between Fingers I–III, moderate between Fingers III and IV, with formula I 2^+^—2^1/2^ II 1^1/2^—3^+^ III 2^+^—1^3/4^ IV. Prepollex concealed; subarticular tubercles round, faint; few small supernumerary tubercles present, palmar tubercle round, of moderate size and difficult to see, thenar tubercle ovoid; nuptial excrescences present as a small pad on external edge of Finger I (Type V). Hind limbs slender, tibia length 59% of SVL; tarsal fold absent; discs of toes round; inner metatarsal tubercle small, outer metatarsal tubercle round, both very difficult to distinguish. Webbing formula of feet: I 1—2^–^ II 1—2^1/2^ III 1—2^1/3^ IV 2^1/4^—1^1/3^ V. In preservative, dorsal skin creamish white, with minute dark lavender melanophores (only visible under the stereomicroscope); dorsal skin shagreen; skin on venter areolate; cloacal opening at level of upper thighs, small and non-enameled cloacal warts present. Parietal peritoneum and pericardium translucent (in life, the red heart is visible ventrally); urinary bladder lacking iridophores; liver, viscera, and tested fully covered by iridophores; kidneys rounded, approximately bean-shaped; liver bulbous.

**Coloration in life.** Dorsal surfaces apple green to yellowish green with diffuse yellow spots; head with light yellow spots that may form an interorbital bar. Melanophores absent from fingers and toes, except Toes IV and V. Ventrally, parietal peritoneum and pericardium transparent, with a red heart always visible. Gall bladder and urinary bladder covered by translucent peritonea; hepatic and visceral peritonea covered by white iridophores; ventral vein red. Iris yellow, with numerous minute lavender spots. Bones white.

**Coloration in preservative**. Dorsal surfaces creamish white dotted with minute dark lavender melanophores; venter uniform cream, with partial translucence; pericardium translucent; visceral peritoneum covered by iridophores. Iris white with minute lavender melanophores. Melanophores absent from hands and feet, except from some few present on dorsal surfaces of Toes IV and V.

**Measurements of holotype**. MZUTI-3299, adult male. SVL = 19.1, femur length = 11.2, tibia length = 11.3, foot length = 8.8, head length = 5.9, head width = 7.4, interorbital distance = 2.2, upper eyelid = 1.5, internarial distance = 1.5, eye diameter = 2.2, tympanum diameter = 0.6, radioulna length = 4.0, hand length = 6.0, Finger I length = 4.4, Finger II length = 3.9, width of disc of Finger III = 0.9.

**Natural History.** At Bosque Protector Los Cedros, individuals were found on the underside of riparian leaves 1–5 m above stream level during the months of November and March. At Río Manduriacu Reserve, during the rainy season (February), a male was found on the underside of a leaf 6 m above a stream; the male was calling next to an egg clutch. At the same reserve, metamorphs have been found perched on leaves 50–150 cm above streams in October and November.

**Figure 11:**
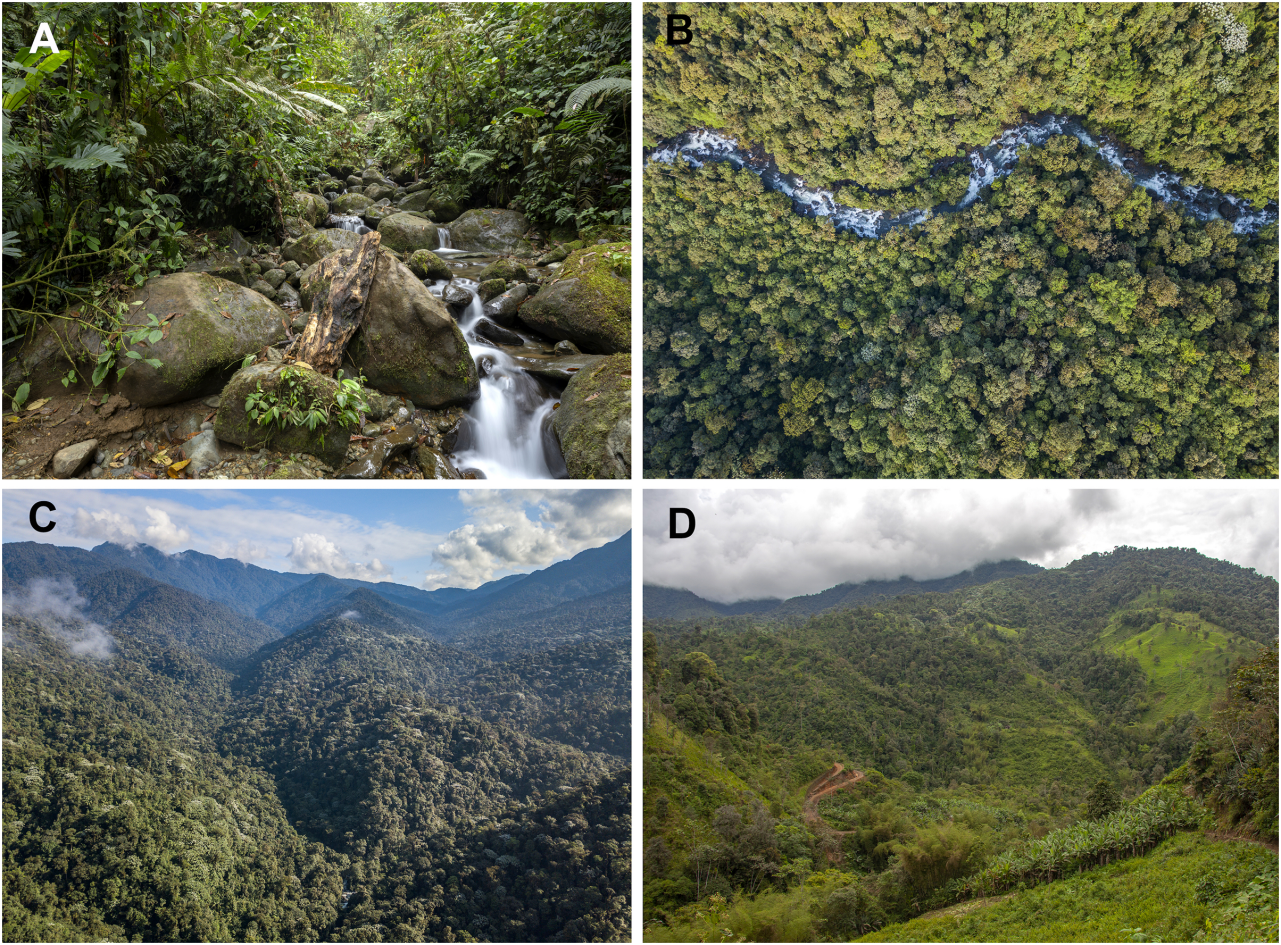
Habitat of *Hyalinobatrachium nouns* sp. nov. (A) Tributary of the Manduriacu River, Río Manduriacu Reserve, Imbabura Province, Ecuador. (B) Tributary of the Manduriacu River, Río Manduriacu Reserve, Imbabura Province, Ecuador. (C) Río Manduriacu Reserve, Imbabura Province, Ecuador. (D) Habitat loss in the vicinity of Los Cedros Reserve, Imbabura Province, Ecuador. *Photos by Jaime Culebras*.

**Distribution.** *Hyalinobatrachium nouns* sp. nov. is only known from Río Manduriacu Reserve and Bosque Protector Los Cedros at elevations of 1,177–1,420 m a.s.l. The reserves are located adjacent to one another and are situated within the Toisán Mountain Range, Imbabura Province, Ecuador (Fig. 10), and protect premontane wet tropical forest and cloud forest (Fig. 11) in an area where illegal deforestation and mining are constant threats (see Discussion).

**Evolutionary relationships**. Our phylogenetic analyses (Fig. 2) place *Hyalinobatrachium nouns* sp. nov. as sister to a clade formed by *H. mashpi* sp. nov. and unidentified haplotypes from the Colombian Andes (MAR 2147, 2222). However, this relationship has low support (bootstrap support = 60). Other closely related taxa are endemic to Central America: *H. vireovittatum* and *H. talamancae* (Fig. 2).

**Etymology.** The specific epithet honors Nouns DAO, a global decentralized autonomous organization (“DAO”) composed of owners of Nouns characters, which are digital art creations that live on the blockchain. The mission of Nouns DAO is to promote and build the Nouns brand throughout the physical and digital world. One of the ways Nouns DAO accomplishes this is by building public works and funding philanthropic projects that support the wonder of nature.

**Conservation status.** We recommend that *Hyalinobatrachium nouns* sp. nov. be listed as Endangered, following IUCN (2012) criteria B1ab(iii): extent of occurrence estimated to be less than 5,000 km^2^; known to exist at no more than five localities; and continuing decline, observed, inferred or projected, in area, extent, and/or quality of habitat. The main threats for this species are habitat loss and contamination due to cattle ranching, agriculture, and mining activities (see below).

## Discussion

**Hidden diversity in the Andes.** The striking homogeneity exhibited by glassfrog in the genus *Hyalinobatrachium* (*sensu* Guayasamin et al., 2009) probably is related to evolutive success of traits such as color pattern (related to camouflage) and reproductive strategies (*e.g*., breeding associated with streams, eggs placed on underside of leaves, extended parental care). Morphological similarity is also expected among closely related glassfrogs because they mainly speciate by allopatry (Hutter, Guayasamin & Wiens, 2013; Castroviejo-Fisher et al., 2014; Guayasamin et al., 2020), retaining the ancestral ecological niche (Wiens, 2004; Hutter, Guayasamin & Wiens, 2013). Therefore, considering morphological traits alone is likely to provide an underestimation of the true species richness within the genus. Congruently, vocalizations and molecular data have been shown to be robust tools to reveal morphologically cryptic taxa in Centrolenidae, as shown herein and previous studies (Castroviejo-Fisher et al., 2011; Hutter & Guayasamin, 2012; Twomey, Delia & Castroviejo-Fisher, 2014; Guayasamin et al., 2020; Escalona-Sulbarán et al., 2019).

The topographical complexity of the Andes, with numerous pronounced river valleys, has favored population structure within species and, ultimately, speciation (Gentry, 1982; Lynch & Duellman, 1997; Madriñán, Cortés & Richardson, 2013; Pérez-Escobar et al., 2017; Polato et al., 2018; Guayasamin et al., 2017b, 2020). Our study provides additional evidence of the biological uniqueness within the Toisán mountain range, which is separated from the western Andes by the Intag-Guayllabamba river valley in the south and the Mira river valley in the north. These valleys seem to be an important dispersal barrier; as a consequence, several anuran sister species are found in the Toisán mountain range and the nearby western Andes: (i) *Hyalinobatrachium nouns* sp. nov. *+ H. mashpi* sp. nov., (ii) *Noblella worleyae + N. mindo* (Reyes-Puig et al., 2021), (iii) *Pristimantis cedros + P. pahuma* (Hutter & Guayasamin, 2015), (iv) *Hyloscirtus princecharlesi + H. ptychodactylus* (Coloma et al., 2012), and (v) genetically differentiated populations of *P. mutabilis* (Guayasamin et al., 2015). The high levels of endemism exhibited by amphibians in the Toisán mountain range likely also apply to other taxa with limited dispersal abilities (*e.g*., flightless invertebrates and small mammals). The two new glassfrog species described herein, although inhabiting forests that are only 18.9 km apart (Fig. 10), have a considerable genetic distance (4.6–4.7%), which is much higher that the intraspecific variation observed in the family, even in species with broad distributional ranges (<3%; Castroviejo-Fisher et al., 2011; Guayasamin et al., 2020).

An unexpected result from our study is that the calls of the sister species *Hyalinobatrachium aureoguttaum* and *H. valerioi* are very similar (Figs. 6, 7). Given the importance of calls in species recognition (Wells, 2010), two scenarios explain the observed data: (i) the two species are fully allopatric and the ancestral call traits have been retained, or (ii) the two species actually represent one evolutionary lineage. Based on our current dataset, we tend to favor the first hypothesis, because there are color (Figs. 3–5) and genetic differences (Fig. 1) between *H. aureoguttaum* and *H. valerioi*. Nevertheless, full clarification would require more sampling (especially in Colombia) and studies in potential contact areas. Finally, within lowland populations of *H. aureguttatum* in Ecuador, we found two clades (Fig. 2); further analyses should determine if these genetic differences are the result of different evolutionary trajectories or retained ancestral polymorphisms (Nichols, 2001).

**Amphibians are the most threatened Andean vertebrates.** Amphibian diversity and endemicity are particularly accentuated in the Andes––roughly 70% of the 1,120 reported species are endemic (CEPF, 2021). The Andes also boasts the highest rate of new amphibian species discoveries of any biogeographic region in South America (Vasconcelos et al., 2019; Womack et al., 2021). Yet, amphibians are particularly susceptible to anthropogenic impacts (Duellman & Trueb, 1994; Lips et al., 2006; Pounds et al., 2006; Scheele et al., 2019), which are immense in the Andes. Currently, only 8% of Andean amphibian species are well-protected (Bax & Francesconi, 2019). An array of human pressures continues to diminish the integrity of Andean terrestrial and freshwater ecosystems (Myers et al., 2000; Knee & Encalada, 2014; Roy et al., 2018; Bax & Francesconi, 2019; CEPF, 2021; Torremorell et al., 2021). As a result, taxonomic groups such as glassfrogs—where a majority of members are endemic to the Tropical Andes, and individual species often have highly restricted distributions—are especially at risk of population declines and extinction (Aguilar et al., 2012; Guayasamin et al., 2019b, 2020; Ortega-Andrade et al., 2021).

Baseline data for amphibians and many Andean taxa—if not most—do not exist. It is therefore difficult to fully appreciate the potential extent of regional biodiversity loss if human landscape modification continues without the implementation of effective mitigation measures (Moura & Jetz, 2021; Pérez-Escobar et al., 2022). Although many tropical areas lack the resources necessary to establish and manage protected areas (Lessmann et al., 2016), the presence of community or non-governmental nature reserves in the Andes can play a crucial role in the protection of amphibians and other threatened species. Notably, our records of *Hyalinobatrachium nouns* sp. nov. were all collected within the boundaries of mining concessions (*i.e*., Reserva Los Cedros and Río Manduriacu Reserve), and records for *H. mashpi* sp. nov. are either within or adjacent to mining concessions (Roy et al., 2018).

Given the plethora of evidence that supports the importance of biodiversity of the Andean region, the decision by the last governments (2007 to present) to encourage large-scale mining operations throughout Andean Ecuador is alarming. Nonetheless, communities in the Intag-Toisán Region and Chocó Andino of northwest Ecuador have demonstrated how unified action, voting for local politicians who support and legislate environmental policies, and partnering with a diverse network of NGOs can result in the ability to meaningfully contest the progression of mining in and around their territories (Avci & Fernández-Salvador, 2016; Roy et al., 2018: Guayasamin et al., 2019b, 2021; Freile et al., 2020). Los Cedros Reserve has become a landmark legal case premised on the rights of nature; the recent ruling by Constitutional Court of Ecuador in favor of Los Cedros opens up the possibility of a domino effect favoring biodiverse areas in the Ecuadorian Andes (Guayasamin et al., 2021).

## Supplemental Information

10.7717/peerj.13109/supp-1 Supplemental Information 1 Museum specimens examined during this study.

10.7717/peerj.13109/supp-2 Supplemental Information 2 *Hyalinobatrachium* species names, museum numbers, genbank codes, and localities for samples sequenced in this study.

10.7717/peerj.13109/supp-3 Supplemental Information 3 Sequences generated in this study. Mitochondrial (16S) sequences will be accessioned in GenBank upon acceptance of this manuscript. The file is in FASTA format.

## Additional Information and Declarations

### Field Study Permissions

The following information was supplied relating to field study approvals (*i.e*., approving body and any reference numbers): Research was conducted under permits MAE-DNB-CM-2015-0017, 019-2018-IC-FAU-DNB/MAE, and MAE-DNB-CM-2018-0105, issued by the Ministerio del Ambiente del Ecuador. Access to field sites was granted by Mashpi Reserve and Fundación Ecominga.

### Data Availability

The following information was supplied regarding data availability: The sequences are available at GenBank: OK383415 to OK383437.

### New Species Registration

The following information was supplied regarding the registration of a newly described species: Publication LSID: urn:lsid:zoobank.org:pub:0C4888D5-2DB9-4421-A96E-7E41C17EC82F. Hyalinobatrachium mashpi, LSID: urn:lsid:zoobank.org:0815B7E6-33FB-42D9-A367-4FB50885C256. Hyalinobatrachium nouns, LSID: urn:lsid:zoobank.org:1A908651-9A82-4DCA-9960-E8DC525F5ADF.
