# Supplementary material for: Two new glassfrogs (Centrolenidae: Hyalinobatrachium) from Ecuador, with comments on the endangered biodiversity of the Andes
Source: PeerJ. 2022 Mar 18;10:e13109. doi: 10.7717/peerj.13109 (PMC8935995; doi:10.7717/peerj.13109)
Supplement: Supplemental Information 1 [file peerj-10-13109-s001.docx]

**Two New Glassfrogs (Centrolenidae: *Hyalinobatrachium)* from Ecuador, with comments on the Endangered Biodiversity of the Andes**

Juan M. Guayasamin, Rebecca M. Brunner, Anyelet Valencia-Aguilar, Daniela Franco-Mena, Eva Ringler, Anderson F. Medina, Carlos Morochz, Lucas Bustamante, Ross J. Maynard, Jaime Culebras

**Appendix S1. Examined Specimens**

*Hyalinobatrachium adespinosai:* Ecuador: *Provincia de Tungurahua:* San Jacinto River (1.3447 S, 78.1814 W; 1795 m asl), ZSFQ 1647–48, 1650–52 (type series).

*Hyalinobatrachium aureoguttatum:* Ecuador: *Provincia de Pichincha:* Reserva Mashpi, Río Malimpia (0.1705 N, 78.888 W; 721–723 m), JMG 2000–2004. *Provincia de Esmeraldas:* Hacienda del señor García (0.51197 N, 79.1343 W; 369–457 m), ZSFQ 1532–1539. Stream affluent of the Río Durango (1.05 N, 78.6167 W; 100–150 m), QCAZ 27429, 6302, 6303, 6441–42, 28802; 2 km E of San Francisco (1.0872 N, 78.6905 W; 60–80 m), on the San Francisco-Durango road, QCAZ 32101–02, 32105, 32129, 32132–33; Río Quingue, nearby Caimito (0.72096 S, 80.09117 W, 47 m), QCAZ 37306. *Provincia de Imbabura:* 6 km SE of Lita (0.79 N, 78.43 W; 600 m), QCAZ 4323. Colombia: *Departamento del Chocó:* Municipio El Carmen de Atrato, km 23 on road El Carmen–Quibdó (5°47' N; 76°20' W, 1030 m), ICN 17507, 17509–10, 17512; km 44 on road El Carmen–Quibdó, 630 m, ICN 17252–54, 17515–16; km 53 on road El Carmen–Quibdó, 420 m, ICN 17248, 17257, 17260, 17262, 17266–67, 17520–21, 17525, 17527–28, 17531–34, 17536–37.

*Hyalinobatrachium valerioi:* Costa Rica: *Puntarenas:* Rincon de Osa, ca. 2.5 km SW of, Osa Tropical Science Center, Quebrada McDiarmid, 30 m, USNM 219398–417.

*Hyalinobatrachium vireovittatum:* Costa Rica: *Provincia de San José:* 16 km SW San Isidro de El General on the road to Dominical, 880 m, LACM 75141 (holotype).

*Hyalinobatrachium talamancae:* Panamá: *Provincia de Coclé:* Parque Nacional General Omar Torrijos, 650–850 m, USNM 57213.
