# Supplementary material for: Two new glassfrogs (Centrolenidae: Hyalinobatrachium) from Ecuador, with comments on the endangered biodiversity of the Andes
Source: PeerJ. 2022 Mar 18;10:e13109. doi: 10.7717/peerj.13109 (PMC8935995; doi:10.7717/peerj.13109)
Supplement: Supplemental Information 2 [file peerj-10-13109-s002.docx]

**Two New Glassfrogs (Centrolenidae: *Hyalinobatrachium)* from Ecuador, with comments on the Endangered Biodiversity of the Andes**

Juan M. Guayasamin, Rebecca M. Brunner, Anyelet Valencia-Aguilar, Daniela Franco-Mena, Eva Ringler, Anderson F. Medina, Carlos Morochz,

Lucas Bustamante, Ross J. Maynard, Jaime Culebras.

**Supplementary Material 2.** *Hyalinobatrachium* species names, museum numbers, genbank codes, and localities for samples sequenced in this study.

| **Species** | **Voucher number** | **Genbank code (16S)** | **Locality** | **Latitude** | **Longitude** | **Elevation (m)** |
| --- | --- | --- | --- | --- | --- | --- |
| *H. aureoguttatum* | ZSFQ 1532 | OK383415 | Ecuador: Esmeraldas Province: private forest. | 0.511972 N | 79.1343 | 369–457 |
| *H. aureoguttatum* | ZSFQ 1533 | OK383416 | Ecuador: Esmeraldas Province: private forest. | 0.511972 N | 79.1343 | 369–457 |
| *H. aureoguttatum* | ZSFQ 1535 | OK383417 | Ecuador: Esmeraldas Province: private forest. | 0.511972 N | 79.1343 | 369–457 |
| *H. aureoguttatum* | ZSFQ 1536 | OK383418 | Ecuador: Esmeraldas Province: private forest. | 0.511972 N | 79.1343 | 369–457 |
| *H. aureoguttatum* | ZSFQ 1537 | OK383419 | Ecuador: Esmeraldas Province: private forest. | 0.511972 N | 79.1343 | 369–457 |
| *H. aureoguttatum* | ZSFQ 1539 | OK383420 | Ecuador: Esmeraldas Province: private forest. | 0.511972 N | 79.1343 | 369–457 |
| *H. aureoguttatum* | ZSFQ 1541 | OK383421 | Ecuador: Esmeraldas Province: private forest. | 0.511972 N | 79.1343 | 369–457 |
| *H. aureoguttatum* | JMG 979 | OK383426 | Ecuador: Pichincha Province: Mashpi Lodge Reserve, Río Malimpia | 0.170459 N | 78.887836 W | 723 |
| *H. aureoguttatum* | MZUTI-4327 | OK383433 | Ecuador: Pichincha Province: Mashpi Lodge Reserve, Río Malimpia | 0.17011 N | 78.88695 W | 721 |
| *H. aureoguttatum* | JMG 2000 | OK383427 | Ecuador: Pichincha Province: Mashpi Lodge Reserve, Río Malimpia | 0.170459 N | 78.887836 W | 723 |
| *H. aureoguttatum* | JMG 2001 | OK383428 | Ecuador: Pichincha Province: Mashpi Lodge Reserve, Río Malimpia | 0.170459 N | 78.887836 W | 723 |
| *H. aureoguttatum* | JMG 2002 | OK383429 | Ecuador: Pichincha Province: Mashpi Lodge Reserve, Río Malimpia | 0.170459 N | 78.887836 W | 723 |
| *H. aureoguttatum* | JMG 2003 | OK383430 | Ecuador: Pichincha Province: Mashpi Lodge Reserve, Río Malimpia | 0.170568 N | 78.887721 W | 721 |
| *H. aureoguttatum* | JMG 2004 | OK383431 | Ecuador: Pichincha Province: Mashpi Lodge Reserve, Río Malimpia | 0.170568 N | 78.887721 W | 721 |
| *H. mashpi* sp. nov. | MASHPI-0302 | OK383425 | Ecuador: Pichincha Province: Mashpi Lodge Reserve, Río San Vicente | 0.16482 N | 78.86545 W | 1101 |
| *H. mashpi* sp. nov. | MZUTI 3921 | OK383432 | Ecuador: Pichincha Province: Mashpi Lodge Reserve, Río Amagusa | 0.15469 N | 78.85322 W | 1137 |
| *H. mashpi* sp. nov. | CJ 11645 | OK383435 | Ecuador: Pichincha Province: Reserva Tayra, tributary of the Mashpi River | 0.11463 N | 78.88307 W | 1126 |
| *H. mashpi* sp. nov. | CJ 11642 | OK383434 | Ecuador: Pichincha Province: Mashpi Lodge Reserve, Río San Vicente | 0.163397 N | 78.86736 W | 1040 |
| *H. mashpi* sp. nov. | CJ 11644 | OK383436 | Ecuador: Pichincha Province: Mashpi Lodge Reserve, Río San Vicente | 0.163397 N | 78.86736 W | 1040 |
| *H. mashpi* sp. nov. | CJ 11643 | OK383437 | Ecuador: Pichincha Province: Mashpi Lodge Reserve, Río San Vicente | 0.163397 N | 78.86736 W | 1040 |
| *H. nouns* sp. nov. | MZUTI 3299 | OK383422 | Ecuador: Imbabura Province: Cordillera de Toisán, Bosque Protector Los Cedros | 0.319 N | 78.781 W | 1420 |
| *H. nouns* sp. nov. | ZSFQ 3906 | OK383423 | Ecuador: Imbabura Province: Cordillera de Toisán, Río Manduriacu Reserve. | 0.3099 N | 78.8567 W | 1202 m |
| *H. nouns* sp. nov. | ZSFQ 0537 | OK383424 | Ecuador: Imbabura Province: Cordillera de Toisán, Río Manduriacu Reserve. | 0.31126 N | 78.8588 W | 1254 m |
